# Sense of Belonging at School and on Social Media in Adolescence: Associations with Educational Achievement and Psychosocial Maladjustment

**DOI:** 10.1007/s10578-023-01516-x

**Published:** 2023-03-15

**Authors:** Matteo Angelo Fabris, Michele Settanni, Claudio Longobardi, Davide Marengo

**Affiliations:** 1 University of Turin, Turin, Italy; 2 Department of Psychology, University of Turin, Via Verdi 10, 10124 Turin, TO Italy

**Keywords:** Sense of belonging, Belonging at school, Belonging online, Psychological maladjustment, Educational achievement, Social media addiction

## Abstract

Belongingness is a key factor in the psychological development and school adjustment of children and adolescents. Going beyond the existing literature, the present study evaluated the association between two sources of belongingness, namely sense of belonging at school (SOBAS) and on social media (SOBOSM), and both psychological maladjustment and educational achievement. Sample consisted of 698 early-to-late adolescents (52.3% female, mean age = 13.79 ± 2.09). Both SOBAS and SOBOSM showed negative direct associations with psychological maladjustment. SOBOSM showed a negative direct association with educational achievement. Social media addiction mediated the links between both SOBAS and SOBOSM and psychological maladjustment and education achievement. Overall, findings suggest that adolescents reporting high sense of belonging both at school and on social media may at lower risk for psychological maladjustment. However, adolescents reporting low SOBAS and high SOBOSM may also be a greater risk of SMA, possibly impacting their psychological health and school adjustement.

**Supplementary Information:**

The online version contains supplementary material available at 10.1007/s10578-023-01516-x.

## Introduction

### Relating a Sense of Belonging at School to Educational Outcomes and Psychological Adjustment

School is an important developmental context for children [1, 2], and a sense of belonging at school (SOBAS) appears to be a crucial factor in promoting more positive academic and developmental outcomes [3, 4]. Despite different terminologies, a sense of belonging refers to “the extent to which students feel personally accepted, respected, included, and supported by others in the school social environment” [5], p. 80). According to the perspectives of self-determination theory and belongingness theory, the need to belong seems to be intrinsic to human beings [6, 7], and a sense of belonging appears to be particularly important for the psychological adaptation of individuals in adolescence [8], which is a transitional period between childhood and adulthood. During this period, there is a strong drive for autonomy, and the peer group becomes the main social group in which to cultivate friendships and find support [9, 10], therefore, although SOBAS is assumed to be an important factor in school adjustment at any age, it may become particularly significant in adolescence. SOBAS tends to meet adolescents’ relatedness needs, promoting more favorable developmental outcomes [4]. For adolescents, SOBAS means being in a safe educational context, characterized by positive relationships, where they can express themselves without fear of rejection [11]. This means that SOBAS contributes to making individuals feel like group members and protects them from the negative effects of social exclusion [11]. SOBAS therefore tends to promote greater psychological adjustment in adolescents, associated with an increase in positive affect and life satisfaction [12], and greater self-esteem and self-concept [4], but is also associated with a reduction in internalizing and externalizing symptoms [13, 14] a decreased sense of loneliness [15], a lower suicide risk [35], and less problematic behaviors, such as delinquency and substance use. Furthermore, SOBAS can reduce the negative impact of victimization experiences on adolescents’ mental health. It may also have an effect on positive academic outcomes through increased use of metacognitive process, peer learning, and adaptive academic help-seeking strategies [16, 17]. Furthermore, SOBAS has been reported to be positively associated with academic achievement and negatively associated with absenteeism and dropout rates [4]. Conversely, low levels of SOBAS are associated with greater procrastination and poor self-regulation [18].

Ultimately, it appears that SOBAS helps adolescents to feel like valuable and important members of their schools, accepted and included by their peers. A sense of belonging thus seems to satisfy adolescents’ needs for relatedness, influencing their psychological well-being and fostering a more positive attitude toward school. It also tends to predict greater psychological adjustment and more favorable school outcomes.

### Belongingness at School, Social Media Use and Addiction, and Their Impact on Educational and Psychological Outcomes

Since belongingness is a basic need of individuals, it is possible that when the need is frustrated, individuals will attempt to compensate for it in other ways. In this vein, social compensation theory suggests that those who suffer from interpersonal problems, or who feel rejected, isolated, or lonely, and therefore experience a diminished sense of relatedness, may attempt to compensate for their social relationship needs and extend their network of relationships in the online world [19]. Evidence has suggested that individuals who have deficient social skills or perceive themselves as isolated and rejected tend to prefer online communication and use social networks to meet their social needs or extend their contact networks O’Day et al., [20]. Adolescents who are socially excluded or have negative relationships with peers are thus known to make greater use of social media [9, 21], which places them at greater risk of developing social media addiction (SMA). Similarly, regarding samples of young adults, some researchers have found a correlation between the need to belong and a risk of SMA [22, 23].

Several terms, such as pathological social media use, problematic or excessive social media use, social media use disorder, and the like have been used to indicate the addictive potential of social media use [24, 25]. According to the behavioral addiction model proposed by Griffiths [26], SMA can be described and defined with respect to the following six dimensions: mood modification (i.e., social media use to promote a positive change in emotional states), salience (i.e., behavioral, cognitive, and emotional preoccupation with social media use), tolerance (i.e., the need to increase social media use over time), withdrawal symptoms (i.e., experiencing unpleasant physical and emotional symptoms when social media use is restricted or stopped), conflict (i.e., problems ensuing because of social media use), and relapse (i.e., addicts quickly reverting back to excessive social media use after a period of abstinence). Between 2.8 and 47% of adolescents are estimated to be at risk of SMA (Banyai et al., 2017), with females generally experiencing a higher risk [27]. Given the ever increasing use of social media and its pervasiveness in everyday life of both adolescents and adults, and the lack of an official diagnosis of SMA, however, in the present study we refrain from pathologizing intensive social media use (see [28] for a comment on this matter) but instead aim at highlighting possibile links between individual tendencies toward problematic social media use and keys developmental outcomes in adolescence.

Several researchers have suggested that SMA is related to a decrease in psychological well-being in adolescents and young adults [29–31]. According to recent cross-cultural research [32, 33], daily use of social media networks for two or more hours per day can foster increased internalizing symptoms and lower academic performance. The relationship between SMA and psychological distress is still not entirely clear, but several hypotheses have been formulated,for example, it is possible that more time spent on social media exposes subjects to greater social comparison, leading to dissatisfaction, envy, and negative feelings that generate psychological distress [34, 35]. Other authors have suggested that SMA may expose adolescents to an increased risk of negative interactions or victimization experiences [30], 2022) that may affect their psychological well-being. Also, especially for those with social skill deficits, the increased frequency of social media use, although aimed at compensating for belongingness needs, makes them feel even more lonely and isolated, decreasing the likelihood of face-to-face interactions and thus reinforcing addictive behavior and its impact on mental health. Increased time spent online may therefore result in fewer opportunities for offline contact and cause a reduction in social opportunities to experience pleasurable and rewarding activities [32, 33, 35]. Finally, excessive use of social media may impact sleep quality and increase sedentary behavior, which may in turn reduce individuals’ psychological and physical well-being (Keles et al., 2020). Also, SMA tends to be associated with lower academic achievement [36, 37], probably due to cognitive resources and time being diverted from study toward social media.

However, notably, research on the relationship between social media use and measures of belongingness and mental health is inconclusive and, particularly regarding the frequency and patterns of social media use, results tend to be mixed [38]. Some factors, such as patterns of social media use (e.g., active use) or some personal characteristics (e.g., extroversion), might be central in mediating the relationship between social media use and academic and psychological outcomes. Evidence has suggested, for example, that social media use is associated with a range of offline interpersonal benefits [39]. Two longitudinal studies [40] reported that greater social media use was associated with greater perceived social support and with higher bridging social capital one year later. However, these studies did not clarify whether perceptions of social support—a construct intimately related to belongingness needs—occurred in the online or offline environment. Considering that the online context can offer cues to build new friendships, contacts, and belong to a group, it is important, in our opinion, to extend current knowledge about perceived belongingness in the online and school contexts to predict developmental and academic outcomes in adolescents.

### Belongingness on Social Media, Social Media Use and Addiction, and Developmental Outcomes

Social media offers important opportunities for adolescents’ social functioning, allowing them to stay connected more easily with their groups of friends or form new relationships on the Web. It is therefore possible that social media, as described above, can offer adolescents spaces and tools to acquire a sense of belonging. However, not all authors agree on the positive impact of social media on belongingness, often taking opposing positions. According to Davis (2012), for example, social media allows adolescents to stay in touch with their friends, thus promoting a sense of connectedness. Some studies have shown that socially anxious and isolated adolescents may make greater use of online communications to reduce their sense of loneliness; meet new people; and share confidences, experiences, and interests, thereby increasing their sense of belonging to a group and preserving a certain level of psychological well-being [41–43]. Other authors, however, have argued that although social media can contribute to the enhancement of belongingness and social connectedness, they may paradoxically increase the risk of ostracization and isolation from peers, causing an increase in psychological distress [44–46]. Depressed young people with social difficulties may prefer online communication and be more passive and less interactive in their use of social media. This preference may be reinforced by the use of the Internet itself, thus increasing feelings of loneliness and distress. Other authors have asserted that adolescents who feel that their sense of belonging is undermined tend to develop more negative affections, stronger feelings of envy, and increased use of social media. Overall, the literature has revealed a complex correlation between social media use, belongingness, and psychological well-being in adolescents. It is possible that social media can provide a space for adolescents to meet belongingness needs, but positive or negative outcomes for individual well-being may depend on how the social is used and the quality of interactions on the Web [45]. Liu and colleagues (2018 reported that when the motivation for using social media is the need to belong, adolescents tend to report increased positive affect. It is possible, however, that feeling connected to others through social media leads individuals to return to social media repeatedly to satisfy this need, fueling the frequency of social media use [45]. Along these lines, evidence has suggested that perceived online support via social networks can predict a greater increase in SMA [47–49]. This seems to contradict the evidence suggesting a negative correlation between perceived offline support and SMA,therefore, it seems important to consider perceived social support simultaneously in online and offline contexts because they are important developmental contexts for adolescents, where they meet and compare themselves with others, seeking social support and shared experiences to foster the perception of a sense of belonging.

### Study Aims

The aim of this study is to extend current knowledge about the possible association between belongingness and both psychological and academic outcomes. In particular, considering that the offline world (school) and the online world (social media) are both important contexts for adolescents’ social functioning, in which they try to satisfy their need to belong, we aim to compare, within the same group of adolescents, the effects that belongingness at school and online separately had on the two areas of developmental outcomes: psychological adjustment and academic grades. It is possible that sense of belonging at school (SOBAS) and sense of belonging on social media (SOBOSM) may have different impacts on youth’s psychological and academic adjustment, but the literature has yet to address this point. To shed light on this, in the present study we aim to examine the roles of SOBAS and SOBOSM, social media use, and SMA in explaining individual differences in students’ educational achievement and levels of psychosocial maladjustment. Secondly, we test the role of social media use and SMA as mediators of the link between SOBAS, SOBOSM, and students’ educational achievement and levels of psychosocial maladjustment. Based on scientific literature, we hypothesize a positive relationship should emerge between SOBAS and reduced psychological maladjustment in adolescence. We also hypothesize the existence of a positive relationship between sense of SOBAS and reduced social media use in adolescents. Following previous findings, we expect that individuals who feel a stronger sense of school belonging may be less likely to show problematic online behaviors, including addiction to excessive social media [50, 51], which in turn is expected to contribute to psychological maladjustment [52]. As such, our additional hypothesis is that the association between SOBAS and psychological maladjustment might be in part mediated by a decrease in SMA. Following previous findings indicating the SMA tends to be associated with lower academic achievement, we also expect an indirect effect might be linking SOBAS to greater academic achievement by means of reduced social media use and SMA [36, 37].

On the other hand, the effects of SOBOSM on psychological maladjustment in adolescence may be more complex. Some studies have found that online social networks can provide a sense of connection and support for adolescents, leading to an increase in well-being [53]. However, as noted about other research has suggested that excessive use of social media can lead to feelings of isolation and social comparison, reducing the overall wellbeing [52]. Overall, we expect that SOBOSM media may improve adolescents’ well-being while also putting them at risk for problematic social media use, which in turn, as noted above, may have negative effects on their academic performance [36, 37].

## Methods

### Sample and Procedure

Participants were recruited among students attending middle and high schools located in in both rural and urban areas of northwestern Italy. The initial sample consisted of 748 students who accessed an online questionnaire, but n = 59 participants were later removed because they failed not complete the questionnaire, including participants failing to agree to participating in the study (n = 11), and participants who failed to answer the questionnaire except for the demographic variable section (n = 39). Eventually, the final sample consisted of 698 students (52.3% female, mean age = 13.79, SD = 2.09), of which 54.6% were early adolescents aged 11–13, and 45.4% were older adolescents aged 14 to 19. The majority of students held an Italian citizenship (93.2%), while a minority of students held a nationality of a country of the European Union (EU) different from Italy (1.4%) or a non-EU citizenship status (5.4%). Please note that we recruited a convenience sample; the distribution of participants’ demographic characteristics is similar to that observed in the Italian student population, albeit a lower percentage of non-Italian students was observed in our study compared with available national data [54].

## Instruments

### Sense of Belonging at School

We administered the Italian version of the Sense of Belonging Scale at School scale (SOBAS) a self-report measure from the OECD-PISA data collection [55], for a cross-national comparison study, see [3]. The scale includes six items sharing a common stem: “When I am at school...” The items were as follows: “I feel like an outsider,” “I make friends easily,” “I feel like I belong,” “I feel awkward and out of place,” “Other students seem to like me,” and “I feel lonely.” Choices were (1) strongly disagree, (2) disagree, (3) neither agree nor disagree, (4) agree, and (5) strongly agree. The item scores were averaged to obtain a total score (mean = 3.267, SD = 0.726, observed range = 1.17–5.00). The reliability was good (α = 0.80).

### Sense of Belonging on Social Media

We created a new measure by adapting the SOBAS scale for the assessment of students’ SOBOSM. The scale included six items sharing a common stem: “When I am on social media...” The items were as follows: “I feel like an outsider,” “I make friends easily,” “I feel like I belong,” “I feel awkward and out of place,” “Other people seem to like me,” and “I feel lonely.” Choices were (1) strongly disagree, (2) disagree, (3) neither agree nor disagree, (4) agree, and (5) strongly agree. The item scores were averaged to obtain a total score. The items, adapted to the social media context, were preliminary validated by administering them to a sample of students (N = 28), that were asked to assess content and face validity. The results of this pretesting was satisfactory, hence we felt entitled to use the newly adapted instrument for our research aims. In the present study, a confirmative factor analysis assuming a single factor provided acceptable fit statistics (χ^2^/df = 4.06; CFI = 0.98; TLI = 0.96; SRMR = 0.02; RMSEA = 0.06) according to criteria proposed by [56], CFI & TLI ≥ 0.95; SRMR < 0.08; RMSEA ≤ 0.06).) The reliability was adequate (α = 0.71).

### Social Media Use

We asked students to report the frequency of their use of the following social media: Facebook, Twitter, YouTube, Tik-Tok, Instagram, Snapchat, Messenger, WhatsApp, and Telegram. Students were also allowed to indicate an additional platform of their choice and provide a rating for the frequency of use. In the absence of information about actual daily use of social media platforms (e.g., digital traces of actual use, or screen time, [57], and in order to contrast the pitfalls of self-report estimates of usage at the hour level (e.g., lack of accuracy leading to overestimation of time spent on social media; [58], we decided to assess social media use over a relatively ample period (a month), distinguishing between participants reporting no use at all to those reporting use on either a monthly, weekly, or daily basis. Albeit providing a low resolution view on adolescents’ recent social media use, this assessment approach has shown stronger correlation with logs of actual technology use [59], than those assessing usage at the hour level [58]. As such, frequency of use was rated according to the following scale: (0) I do not have an account, (1) I have an account, but I never or seldom use it, (2) Once or twice a month, (3) Once or twice a week, (4) Almost every day, (5) Every day. A total score was computed by summing the scores for each platform (mean = 18.267, SD = 7.048, observed range = 0–40).

### Social Media Addiction

We administered an Italian adaptation of Bergen’s Social Media Addiction scale (BSMAS; [27]. BSMAS includes six items that assess six components of addictive social media use: salience, tolerance, mood modification, relapse, withdrawal symptoms, and conflict. Example items are: “How often during the last year have youfelt an urge to use social media more and more?” (Tolerance), “How often during the last year have you used social media in order to forget about personal problems?” (Mood mofication). Participants were asked to report about their experience during the previous 12 months. The items were rated on a five-point scale ranging from (1) very rarely to (5) very often. The item scores were averaged to obtain a total score. The reliability was adequate (α = 0.71).

### Psychosocial Maladjustment

We administered the Italian version [60] of the Strengths and Difficulties Questionnaire (SDQ; [61], which is suitable for early- to mid-adolescent students. The SDQ includes 25 items measuring components of students’ psychological adjustment, each assessed using 5 items: emotional problems (e.g., “I am often unhappy”), conduct problems (e.g., “I am often accused of lying or cheating”), hyperactivity/inattention (e.g., “I find it difficult to concentrate”), peer relationship problems (e.g., “I have one or several friends” [reversed]), and prosocial behavior (e.g., “I try to be nice to other people”). All items were rated using a three-point Likert scale: (0) not true, (1) somewhat true, and (2) certainly true. Responses to all items were summed to create a total score representing a global indicator of students’ psychosocial maladjustment. The reliability was adequate (α = 0.74).

### Educational Achievement

We asked students to report their own levels of educational achievement by stating their most recent grades for each school subject, in a range from 0 to 10 for performance (i.e., the common metric for grades in the Italian education system). The grades were then averaged to obtain an overall measure of students’ current level of educational achievement in school subjects.

### Analytical Strategy

**Fig. 1:**
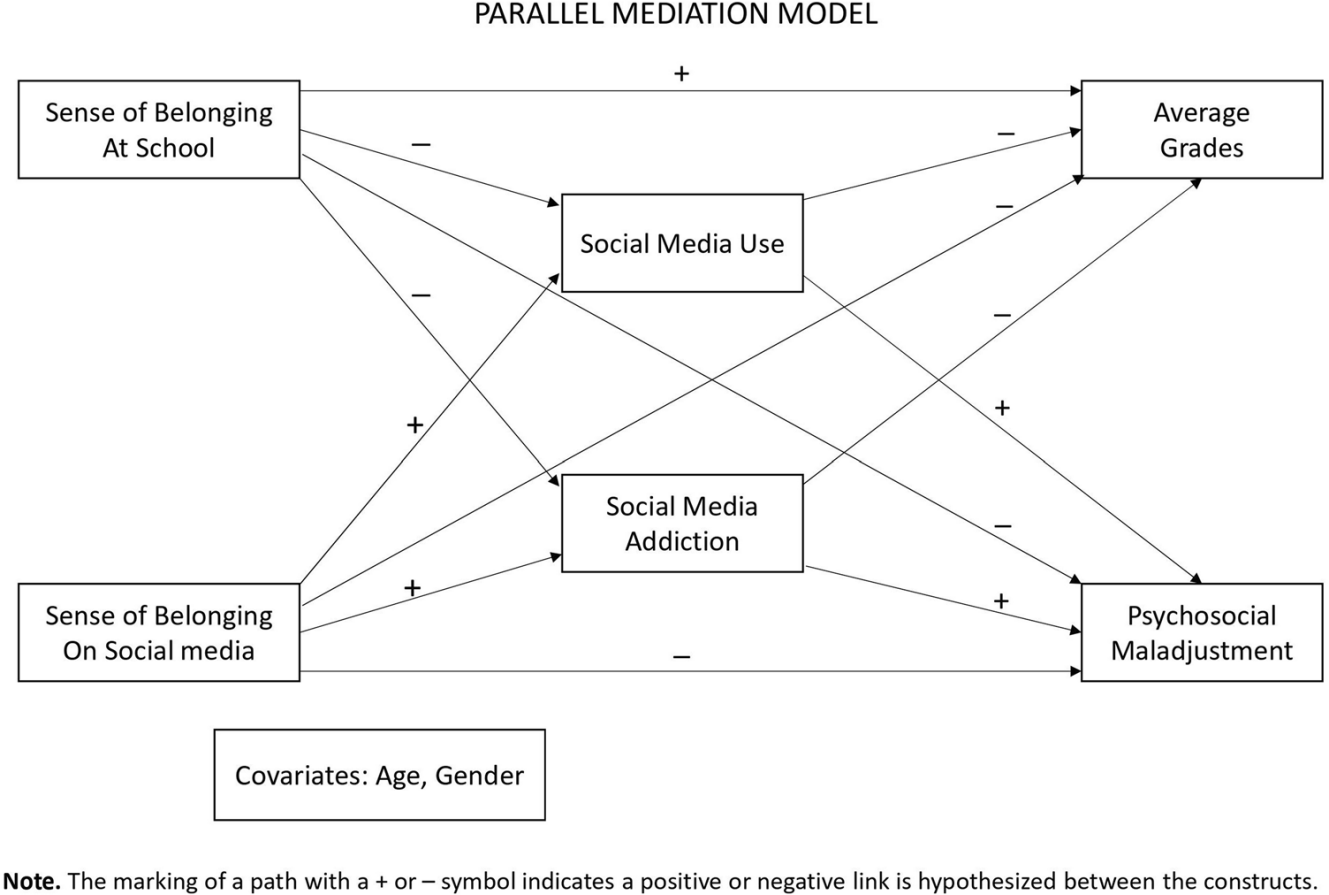
Diagram for the parallel mediation model

First, we computed descriptive statistics (mean and standard deviation for continuous measures, and percentage for binary variables) for all the study measures, and examined Pearson’s correlations between them. Next, we tested the links between students’ SOBAS and SOBOSM and both their school achievements (average grades) and their psychosocial maladjustment (i.e., the SDQ total score). Using a parallel mediation model, we tested the hypothesis that frequency of social media use and current level of SMA might mediate these links. Figure 1 presents a diagram representing the hypothesized direct paths. Path coefficients were estimated using a regression approach and 10,000 bootstrap samples. We followed this approach because use of the bootstrap procedure does not impose strict distributional assumptions on the residuals (as opposed to regular regression analysis), hence allowing for inference even if the errors do not follow a normal distribution or constant error variance [62]. In estimating the path coefficients, we controlled for students’ age and gender. Effects were deemed statistically significant if 95% confidence intervals did not include zero. Analyses were performed using SPSS® 23 and the PROCESS macro, model 4 [63].

## Results

### Correlations Between Study Measures

The results of the correlations between the study measures are shown in Table 1, along with descriptive statistics for all variables. Age had a moderate positive association with overall social media use and a small positive association with SMA. Age also had a small negative correlation with students’ school grades. Being male had a small positive correlation with students’ SOBAS, and small negative correlations with SMA, the average of school grades, and psychosocial maladjustment according to the SQD total score. SOBAS had a small positive correlation with students’ SOBOSM, a small negative correlation with SMA, and a strong negative correlation with psychosocial maladjustment. Moreover, SOBOSM had small positive correlations with both social media use and SMA, and a small negative correlation with school grades and students’ levels of psychosocial maladjustment. Social media use had a moderate positive correlation with SMA and a small positive correlation with students’ psychosocial maladjustment. We also found that social media use was negatively associated with students’ grades, whereas SMA had a small negative correlation with school grades and a moderate positive correlation with students’ psychosocial maladjustment. Finally, school grades had a small negative correlation with students’ psychological maladjustment. The remaining correlations were either non-significant or negligible in size (r < 0.10).

**Table 1:** Correlation between study measures (N = 698)

	Measures	Mean/%	SD	1	2	3	4	5	6	7
1	Age	13.79	2.09							
2	Gender (Female = 0; Male = 1)	47.7%	–	− .086*						
3	Sense of Belonging—At School	3.83	0.73	− .075*	.159**					
4	Sense of Belonging—On Social media	3.75	0.61	.016	.086*	.291**				
5	Social Media Use	18.27	7.05	.415**	− .079*	− .048	.146**			
6	Bergen Social Media Addiction	2.16	0.76	.163**	− .148**	− .188**	.148**	.321**		
7	Average School Grades	6.89	0.85	− .119**	− .152**	.056	− .141**	− .228**	− .205**	
8	SDQ—Total Difficulties	22.21	6.05	.076*	− .190**	− .548**	− .169**	.187**	.397**	− .148**

*p < .05 **p < .01

### Association Between SOBAS and SOBOSM and Students’ Educational Achievement and Psychosocial Maladjustment

**Fig. 2:**
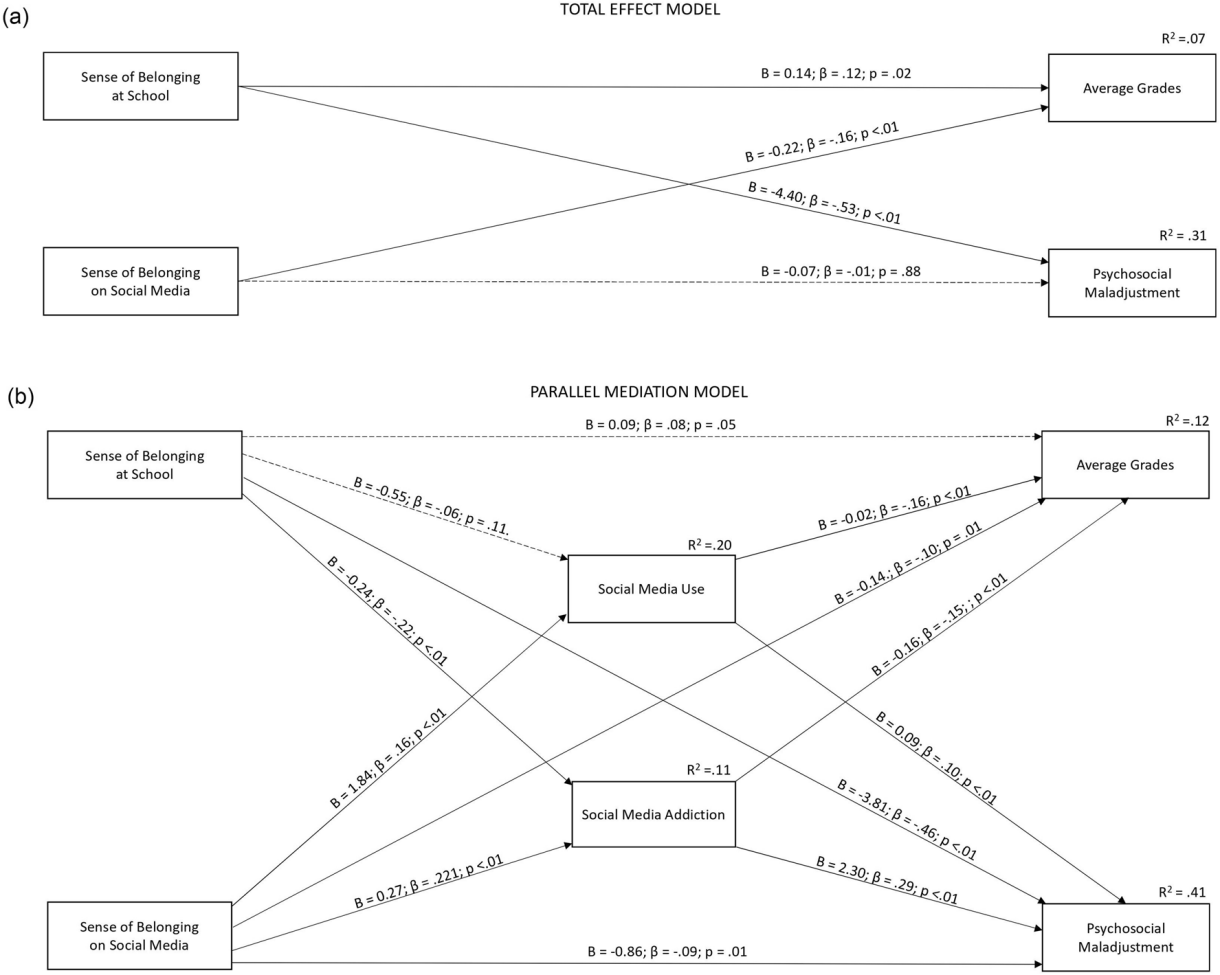
Total Effect and Parallel Mediation Models: Unstandardized and Standardized Regression Coefficients

Figure 2 shows the results of the mediation analyses. Panel A provides a visualization of the path coefficients estimated for the total model, representing the total effects of students’ SOBAS and SOBOSM on both their grades and their psychosocial maladjustment. For ease of visualization, the figure does not provide information about the effects of covariates (i.e., age and gender) and does not include a 95% confidence interval for model parameters based on 10,000 bootstrap samples. This information is reported in the following text. Please note that all model path estimates are also reported in full in the supplementary material, Table S1.

The results indicated that students’ SOBAS was associated with higher grades (B = 0.140, 95% CI [0.051, 0.229], β = 0.120) and lower psychosocial maladjustment (B = − 4.401, 95% CI [− 4.947, − 3.855], β = − 0.528). Moreover, students’ SOBOSM had a negative effect on their grades (B = − 0.220, 95% CI [− 0.324, − 0.116], β = − 0.159), while no association emerged with their psychosocial maladjustment (B = − 0.069, 95% CI − 4.9474, 0.000], β = − 0.007). Finally, age had a negative association with grades (B = − 0.050, 95% CI [− 0.079, − 0.020], β = − 0.122) but no association with psychosocial maladjustment (B = 0.089, 95% CI [− 0.099, 0.261], β = 0.028). Gender (male) had a negative effect on grades (B = − 0.286, 95% CI [− 0.411, − 0.162], β = − 0.168) and on psychological maladjustment (B = − 1.256, 95% CI [− 2.018, − 0.494], β = − 0.104).

Next, Fig. 2 (panel B) provides a visualization of the parallel mediation model. In this model, social media use and SMA were examined as mediators of the effects of students’ SOBAS and SOBOSM on their grades and psychosocial maladjustment. First, we observed that students’ SOBAS had a negative association with their self-reported SMA (B = − 0.235, 95% CI [− 0.319, − 0.152], β = − 0.223), but not with their overall social media use (B = − 0.554, 95% CI [− 1.267, 0.159], β = − 0.057). Furthermore, students’ SOBOSM had a positive association with both SMA (B = 0.275, 95% CI [0.180, 0.374], β = 0.221) and social media use (B = − ì1.84, 95% CI [0.997, 2.702], β = 0.161). Regarding the hypothesized mediators, social media use had a negative association with grades (B = − 0.020, 95% CI [− 0.0304, − 0.0093], β = − 0.163) and a positive association with psychological maladjustment (B = 0.087, 95% CI [0.030, 0.142], β = 0.101). SMA also had a negative association with students’ grades (B = − 0.162, 95% CI [− 0.258, − 0.065], β = − 0.146) and a positive association with psychosocial maladjustment (B = 2.300, 95% CI [1.790, 2.808], β = 0.291). Interestingly, after including social media use and SMA in the model, SOBAS no longer had a direct association with students’ grades (B = 0.091, 95% CI [− 0.002, 0.183], β = 0.078), whereas the negative link with psychosocial maladjustment was retained (B = − 3.812, 95% CI [− 4.359, − 3.275], β = − 0.457). SOBOSM still had a negative effect on students’ grades (B = − 0.139, 95% CI [− 0.248, − 0.030], β = − 0.101) but a new negative effect on their psychosocial maladjustment (B = − 0.860, 95% CI [− 1.449, − 0.217], β = − 0.087).

Next, we examined the indirect effects (see Table 2). Based on 95% confidence intervals (10,000 bootstrap samples), we observed that SOBAS had a negative indirect effect on students’ psychosocial maladjustment through a decrease in SMA (but not social media use). In turn, students’ SOBOSM had a positive indirect effect on psychosocial maladjustment through an increase in social media use and SMA. Regarding students’ grades, we found a positive indirect effect of students’ SOBAS through a decrease in SMA (but not social media use). Finally, SOBOSM had a negative indirect effect on students’ grades through an increase in both social media use and SMA.

**Table 2:** Parallel mediation model: unstandardized effects with 95% confidence interval (10,000 Bootstrap Samples) and standardized indirect effects

Router of indirect effects	Effect	Lower limit	Upper limit	Stand. Effect
Sense of belonging at school → Social Media Addiction → Psychological Maladjustment	− 0.541	− 0.776	− 0.336	− 0.065
Sense of belonging at school → Social Media Use → Psychological Maladjustment	− 0.048	− 0.131	0.124	− 0.006
Sense of belonging on social media → Social Media Addiction → Psychological Maladjustment	0.632	0.397	0.901	0.064
Sense of belonging on social media → Social Media Use → Psychological Maladjustment	0.159	0.048	0.306	0.016
Sense of belonging at school → Social Media Addiction → Average Grades	0.038	0.015	0.065	0.032
Sense of belonging at school → Social Media Use → Average Grades	0.011	− 0.003	0.028	0.009
Sense of belonging on social media → Social Media Addiction → Average Grades	− 0.044	− 0.079	− 0.016	-0.032
Sense of belonging on social Media → Social Media Use → Average Grades	− 0.036	− 0.066	− 0.014	-0.026

Finally, we take a look at the covariates included in the model. Age had no association with psychosocial maladjustment (B = − 0.148, 95% CI [− 0.325, 0.030], β = − 0.051), whereas male gender had a negative association (B = − 0.772, 95% CI [− 1.487, − 0.068], β = − 0.064). Similarly, age had no significant association with students’ grades (B = − 0.015, 95% CI [− 0.047, 0.018], β = − 0.037), but male gender had a negative association (B = − 0.330, 95% CI [− 0.451, − 0.209], β = − 0.194). Age had a positive link with social media use (B = 1.3595, 95% CI [1.126, 1.590], β = 0.404), whereas male gender had no significant association (B = − 0.6951, 95% CI [− 1.634, 0.244], β = − 0.049). Regarding SMA, age had a positive association (B = 0.048, 95% CI [0.022, 0.076], β = 0.132), whereas male gender had a negative association (B = − 0.184, 95% CI [− 0.293, − 0.0739], β = − 0.120).

Interestingly, when comparing the models including covariates and the model with no covariates (not reported in the present manuscript), no relevant emerged in the estimated paths except for the link between SOBAS and social media use, which was associated with significant negative effect in the model including no covariates, but failed to retain significance after inclusion of the covariates in the model.

## Discussion

The aim of this study was to examine the impact that belongingness at school and belongingness on social media had on two general indicators of school adjustment: psychological adjustment and academic achievement. The study took its cue from the theory suggesting that the need to belong is one of the basic human needs and its fulfillment is a factor promoting better developmental outcomes [6, 7]. However, social media has only recently begun to be considered a possible source of belongingness for adolescents. The literature seems to have focused on social media as a means of compensating for deficiencies in satisfaction with a sense of belonging in peer groups (primarily belongingness at school). However, research still scarcely compares the effects that belongingness in offline (school) and online (social media) developmental contexts have, respectively, on different developmental outcomes. Our study aimed to contribute to this direction by analyzing the possible mediating role of social media use and SMA.

In our study, measures of belongingness at school and on social media showed direct and indirect associations with psychological maladjustment and academic achievement. More specifically, SOBAS showed both direct and indirect associaitons with psychological maladjustment, but only an indirect link with academic achievement. In parallel, belongingness on social media showed both direct and indirect associations with psychological maladjustment and academic achievement, respectively. Overall, our data supported the theory suggesting that satisfaction of belongingness tend to be associated with more favorable developmental outcomes. In particular, our findings aligns with a body of evidence suggesting that SOBAS is associated with lower psychological distress [12, 13] and better academic achievement [16, 17]. It is possible that perceptions of better SOBAS are associated with greater perceptions of being welcomed and accepted, eventually leading to the development of a positive social support network and a stronger self-concept, with an impact on an individual’s psychological well-being. Furthermore, several studies have suggested that belongingness at school is associated with a more positive view of school, greater engagement, and a tendency to use help-seeking strategies more frequently [16, 17]. The perception of belongingness could support students’ academic development by providing support for learning activities and reducing distress related to learning challenges. Our study seems to lead in this direction, showing an association between SOBAS and higher levels of academic achievement by way of a reduction in SMA. Moreover. SMA appeared to mediate the relationship between SOBAS and both psychological maladjustment and academic achievement: higher SOBAS was associated with better psychological adjustment and better academic achievement through a reduction of SMA symptoms. These links might be best intepreted in light of social compensation theory, which posits that individuals who experience a lack of social support or belonging in their offline lives (e.g., at school may turn to online platforms, such as social media, which in turn may represent a source of compensation, offering adolescents a space in which to create a network of virtual contacts, increase self-disclosure, and share interests [19, 20]. Some adolescents, particularly those who have had negative experiences of peer relationships or those with poor social skills, may prefer online communication to face-to-face interactions [20], thus, individuals who experience low belongingness in classroom contexts may perceive online communication on social media as fulfilling their need for relatedness, potentially leading to them becoming addicted. In this direction, evidence has suggested that a high need for belongingness is associated with an increased risk of SMA [21, 22]. However, higher levels of SMA tend be associated with increased psychological symptoms [29–31] and reduced academic success [36, 37]. Our data points in this direction. It is possible, therefore, that in order satisfy unmet needs to belong in the school context, adolescents may make greater use of social media, ultimately increasing their risk of SMA. However, increased use of social media can negatively influence students’ psychological health in different ways,for example, it is possible that SMA exposes adolescents to a greater risk of negative interactions or victimization, impacting their health. Also, frequent exposure to social media may paradoxically make individuals feel even more alone and isolated, increasing negative emotions as a result of continuous social confrontation [34, 64]. Moreover, increased use of social media may negatively affect an individual’s life satisfaction, reducing the possibility of establishing social relationships in offline contexts and sharing and experiencing pleasant and rewarding activities, thus increasing the sense of loneliness. Furthermore, as noted above, evidence has suggested that SMA is associated with a decrease in school performance [36, 37]—a claim that our data supported. It is possible that increased SMA demands attention and cognitive resources that could better be devoted to study. Additionally, excessive social media use and SMA can result in school burnout and decrease in school performance in part due an increase in sleep disturbances having a negative impact on academic efficiency [65]. Indicatively, then, our study suggests that high SOBAS might protect pupils’ psychological well-being and their academic performance. In this sense, it is possible that SOBAS may stimulate greater participation in, and satistifaction with educational activities and school life, eventually reducing the risk of SMA and, thereby, improving psychological well-being and academic achievement. Conversely, pupils who perceive lower SOBAS may increase their use of social media, putting them at risk of addiction, with a negative impact on psychological well-being and academic achievement. Our study, however, indicated that high SOBAS, although it appears to be negatively associated with SMA symptoms, is not associated with overall social media use, which is a core feature of SMA. This finding is curious and warrants clarification, particularly in terms of investigating which components of SMA are most related to SOBAS. However, we must bear in mind that social media are particularly widespread among adolescents and are not only used to make new contacts but also to maintain current relationships, including with peers at school [66, 67]. It is therefore possible that, when social media is used to maintain the social contacts developed at school, belongingness at school is not particularly predictive of overall social media use. However, further studies may better clarify this finding.

With regard to SOBOSM, our data suggested a possible direct effect of SOBOSM in decreasing levels of psychological maladjustment. The literature on social media use, sense of belonging, and psychological adjustment is expanding and presenting mixed results [45]. Again, our data seemed to agree with compensation theory, according to which social media can provide a context in which adolescents can develop a sense of belonging, meaning that adolescents can use social media to extend their contact networks, make new friends, share interests, and find social support [41, 42]. Some authors have pointed out that social media can also have a positive impact on offline interpersonal relationships [39]. Overall, then, our data seem to align with the idea that social media may offer support for adolescents to feel connected with others and receive greater social support. However, our data indicated a possible negative effect of SOBOSM on psychological well-being and academic achievement by meanns of heightened social media use and SMA, which appeared to be mediating factors. Our data agree with previous studies claiming an association between perceived online social support and higher levels of SMA [47–49]. It is possible that adolescents who feel that their belonging needs are frustrated and who have difficulties in interpersonal relationships or deficient social skills in the offline world will attempt to meet their social needs through social media. Lonely adolescents with poor social skills may prefer online relationships and communication, thus replacing face-to-face interactions [20]. The gratification of belongingness needs through social media, for these subjects, may not only reinforce but also increase the use of social media, placing them at heightened risk of developing SMA. Adolescents may attempt to compensate for their lack of belongingness through social media. In particular, it is likely that for adolescents who are isolated, rejected, or have poor social skills, the online experience may satisfy the need to belong, leading to increased and continuous use of social media and thus stimulating the development of an addiction. Anxious adolescents or adolescents with social skill deficits may also have emotional regulation deficits, and the SMA may constitute a dysfunctional attempt to regulate their emotions. The frequent use of social media and the risk of SMA could therefore represent a continuous attempt to maintain satisfaction of the needs for connection and relationships with others, frustrated in offline relationships but satisfied in the online world. However, as noted previously, heightened social media use and SMA tend to be associated with psychological maladjustment and poorer academic achievement, negatively affecting adolescents’ psychosocial adjustment. In particular, several studies have suggested that in adolescence and young adulthood, SMA tends to be a risk factor for psychological well-being [29–33] and academic success [36, 37]. Overall, our data suggested that high levels of belongingness on social media may have a direct effect on adolescent psychological well-being, but also indirect negative effects. Arguably, the motivations for adolescents’ attempts to approach others on social media, and their personality characteristics (such as social skills and extroversion), may better explain the relationship between belongingness on social media and social media use, including the risk of SMA, and in turn their possible beneficial or negative impacts on psychological adjustment and school performance. Additionally, although referrring to social compensation theory has been useful in explaining some of the links emerging from the present study, it appears to have limitations when it comes to understanding the relationship between school belonging and social media addiction, suggesting the links between these constructs may be more complex. In particular, one could argue that social media addiction may also act as a barrier to school belonging by deviating students from engaging in school-related activities and in-person relationships, and thus possibly contributing to social isolation and feelings of disconnection [45], which may exacerbate existing feelings of not belonging. Researchers should therefore explore more complex models in future studies, including longitudinal models, to provide additional clarity on the links between these constructs.

In conclusion, school and the online environment are contexts that offer children opportunities to form relationships with others and develop a greater sense of inclusion and connectedness. The perception of belongingness at school tends to be associated, either directly or indirectly, with better developmental outcomes, including greater psychological adjustment and more positive academic outcomes. Social media belongingness may also stimulate greater psychological well-being but not better academic performance. It is possible that, beyond self-reported psychological well-being, greater SOBOSM leads adolescents to experience the online environment more fully, with a consequent decrease in the investment of attentional energy and resources in school activities, and procrastination [36]. Also, adolescents may find the online world more rewarding in view of the academic difficulties they may face in school contexts, leading to lower academic performance. The relationship between belongingness on social media and measures of psychological well-being, however, appears to be complex, and excessive use of social media may mediate a negative relationship between belongingness on social media and psychological well-being. Future studies could explore this aspect further and attempt to understand the factors that may explain the relationship between belongingness on social media and excessive use of social media, and its ultimate impact on adolescents’ emotional and interpersonal functioning. Based on our findings, we believe that future research should increasingly consider belongingness by examining different developmental contexts, such as school and social media. Our research suggested that belongingness on social media and belongingness at school may contribute to adolescent adjustment, potentially affecting different areas of adolescent functioning through different mechanisms. In this sense, SOBAS seems to influence adolescents’ psychological well-being and, indirectly, their academic achievement, offering protection against excessive use of social media. SOBOSM is associated with lower academic achievement and, by means of heigthened SMA, which in turn may exert a negative influence on adolescents’ psychological health.

Our results must be considered in light of the various limitations of the study. Indeed, the cross-sectional nature of the study precluded us from observing causal links between the variables investigated; therefore, longitudinal studies could clarify how the relationships between constructs vary over time and with causality. Also, the use of self-report instruments could have led to social desirability bias or deficits in memory or text comprehension. In particular, findings indicate a general lack of validity of self-report measures assessing frequency of technology use, in particular when a retrospective assessment of the amount of time adolescents spend on social media on a daily basis is of interest. There is some evidence that self-report estimates of daily use (e.g., in hours) tend to deviate significantly from actual use [68, 69]), and that accuracy appears to dependent on both individual characteristics [70] the albeit a debate exists on this matter, see [71] and the specific social media platform: adolescents are more accurate in assessing time spent on platforms when these are used in a a more countinous way (Instagram) than on platforms that are used more sparsely (e.g., Snapchat, WhatsApp) [58]. Future studies should consider collecting objective information about actual technology use (i.e., digital traces of smartphone and social media use; [66, 67] instead of relying on self reports, as done in the present study. Still concerning the employed measures, the present study included a newly devised scale assessing the SOBOSM construct: although this measure showed adequate functioning in a small pre-test evaluation and showed acceptable psychometric properties in the present study, a more comprehensive validation of this measure is required to ensure the validity robustness of our findings. Finally, although the sample was large, it was a convenience sample that cannot be defined as representative of the Italian adolescent population. This limits the generalizability of the results and, therefore, future studies should recruit larger and more representative samples, as well as test the validity of the model in diverse cultures.

## Summary

In a sample of early-to-late adolescents, we investigated the associations between two sources of belongingness, namely sense of belonging at school and on social media, and respectively psychological maladjustment and educational achievement. Sense of belonging at school and on social media were both found to be associated with lowered psychological maladjustment. However, sense of belonging on social media was found to be negatively associated with educational achievement. Of note, social media addiction was found to mediate the links between both sources of sense of belonging and respectively psychological maladjustment and educational achievement. Overall, our findings suggest that adolescents with high sense of belonging at school and on social media are less likely to experience psychological maladjustment, but those experiencing low sense of belonging at school and a high sense of belonging on social media may be at greater risk of social media addiction, which in turn may negatively affect their psychosologial and educational adjustment.

## Supplementary Information

Below is the link to the electronic supplementary material.

Supplementary file1 (DOCX 15 KB)

## Data Availability

The data that support the findings of this study are available from the corresponding author upon reasonable request.
