# Supplementary material for: Sense of Belonging at School and on Social Media in Adolescence: Associations with Educational Achievement and Psychosocial Maladjustment
Source: Child Psychiatry Hum Dev. 2023 Mar 15;55(6):1620–33. doi: 10.1007/s10578-023-01516-x (PMC11485285; doi:10.1007/s10578-023-01516-x)
Supplement: Supplementary file 1 — Supplementary file1 (DOCX 15 KB) [file 10578_2023_1516_MOESM1_ESM.docx]

| Dependent variable | Effect | Effect | SE | LLCI | ULCI | p | Std. Effect |
| --- | --- | --- | --- | --- | --- | --- | --- |
| Social media addiction | Age | 0.05 | 0.01 | 0.02 | 0.08 | <.01 | 0.13 |
|  | Gender | -0.18 | 0.06 | -0.29 | -0.07 | <.01 | -0.12 |
|  | Sense of Belonging on Social Media | 0.27 | 0.05 | 0.18 | 0.37 | <.01 | 0.22 |
|  | Sense of Belonging at School | -0.24 | 0.04 | -0.32 | -0.15 | <.01 | -0.22 |
|  |  |  |  |  |  |  |  |
| Social media use | Age | 1.36 | 0.12 | 1.13 | 1.59 | <.01 | 0.40 |
|  | Gender | -0.70 | 0.48 | -1.63 | 0.24 | .15 | -0.05 |
|  | Sense of Belonging on Social Media | 1.84 | 0.43 | 1.00 | 2.69 | <.01 | 0.16 |
|  | Sense of Belonging at School | -0.55 | 0.36 | -1.24 | 0.16 | .11 | -0.06 |
|  |  |  |  |  |  |  |  |
| Educational achievement | Age | -0.01 | 0.02 | -0.05 | 0.02 | .35 | -0.04 |
|  | Gender | -0.33 | 0.06 | -0.45 | -0.21 | <.01 | -0.19 |
|  | Social Media Addiction | -0.16 | 0.05 | -0.26 | -0.06 | <.01 | -0.15 |
|  | Social Media Use | -0.02 | 0.01 | -0.03 | -0.01 | <.01 | -0.16 |
|  | Sense of Belonging on Social Media | -0.14 | 0.06 | -0.25 | -0.03 | .01 | -0.10 |
|  | Sense of Belonging at School | 0.09 | 0.05 | 0.00 | 0.19 | .05 | 0.08 |
|  |  |  |  |  |  |  |  |
| Psychological maladjustment | Age | -0.15 | 0.09 | -0.32 | 0.02 | .11 | -0.05 |
|  | Gender | -0.77 | 0.37 | -1.49 | -0.05 | .03 | -0.06 |
|  | Social Media Addiction | 2.30 | 0.25 | 1.80 | 2.80 | <.01 | 0.29 |
|  | Social Media Use | 0.09 | 0.03 | 0.03 | 0.14 | <.01 | 0.10 |
|  | Sense of Belonging on Social Media | -0.86 | 0.32 | -1.47 | -0.22 | .01 | -0.09 |
|  | Sense of Belonging at School | -3.81 | 0.28 | -4.36 | -3.27 | <.01 | -0.46 |

**Supplementary material**

Table S1. Model effects with 95% confidence interval (10000 bootstrap samples)
